# *BRCA* Screening and Identification of a Common Haplotype in the Jewish Community of Rome Reveal a Founder Effect for the c.7007G>C, p. (Arg2336Pro) *BRCA2* Variant

**DOI:** 10.3390/cancers17121906

**Published:** 2025-06-08

**Authors:** Laura De Marchis, Alain Jonathan Gelibter, Giulia Mammone, Raffaele Angelo Madaio, Paolo Aretini, Maria De Bonis, Stefania Zampatti, Cristina Peconi, Daniele Guadagnolo, Annarita Vestri, Antonio Pizzuti, Emiliano Giardina, Ettore Domenico Capoluongo, Angelo Minucci

**Affiliations:** 1 Division of Medical Oncology, Department of Radiological, Oncological and Pathological Sciences, Sapienza University of Rome, 00185 Rome, Italy; 2 Medical Oncology Unit B, Department of Hematology, Oncology and Dermatology, Policlinico Umberto I, 00161 Rome, Italy; alain.gelibter@uniroma1.it; 3 Division of Medical and Translational Oncology, Azienda Ospedaliera Santa Maria, 05100 Terni, Italy; mammone.giulia@gmail.com; 4 Oncology Unit, Jewish Hospital of Rome, 00148 Rome, Italy; r.madaio@ospedaleisraelitico.it; 5 Fondazione Pisana per la Scienza, 56017 Pisa, Italy; p.aretini@fpscience.it; 6 Departmental Unit of Molecular and Genomic Diagnostics, Fondazione Policlinico Gemelli IRCCS, 00168 Rome, Italy; maria.debonis@policlinicogemelli.it; 7 Genomic Medicine Laboratory, UILDM IRCCS Santa Lucia Foundation, 00179 Rome, Italyc.peconi@hsantalucia.it (C.P.); angelo.minucci@policlinicogemelli.it (A.M.); 8 Department of Molecular Medicine, Sapienza University of Rome, 00185 Rome, Italy; daniele.guadagnolo@uniroma1.it (D.G.); emiliano.giardina@uniroma2.it (E.G.); 9 Department of Public Health and Infectious Disease, Sapienza University of Rome, 00161 Rome, Italy; annarita.vestri@uniroma1.it; 10 Department of Experimental Medicine, Sapienza University of Rome, 00161 Rome, Italy; antonio.pizzuti@uniroma1.it; 11 Casa Sollievo della Sofferenza Hospital, IRCCS, 71013 San Giovanni Rotondo, Italy; 12 Dipartimento di Eccellenza in Medicina Molecolare e Biotecnologie Mediche, Università Federico II, 80146 Naples, Italy; edotto70@gmail.com; 13 Unità Operativa Complessa di Patologia Clinica, Ospedale S. Giovanni Addolorata, 00184 Rome, Italy; 14 Genomics Research Core Facility, Gemelli Science and Technology Park (GSTeP), Fondazione Policlinico Universitario Agostino Gemelli IRCCS, 00168 Rome, Italy

**Keywords:** *BRCA1/2* genes, Jewish communities, founder mutation, haplotype, ethnical minorities, c.7007G>C variant

## Abstract

Background/Objectives: Cancer risk-reducing strategies in Ashkenazi women carrying founder variants have a cost-effective effect on reducing cancer morbidity and mortality. The British and US guidelines recommend *BRCA1/2* (*BRCA*) screening among Ashkenazi Jewish people to identify high-risk individuals. *BRCA* status has not been investigated yet in the Jewish community of Rome. Methods: Patients were selected from the Family Cancer Clinic of the Umberto I University Hospital of Rome, and 38 unrelated families (28 of Roman Jewish and 10 of Libyan Jewish origin) were enrolled, comprising 44 subjects diagnosed with breast and/or ovarian cancer. Genetic counseling and germline *BRCA* testing were conducted. Haplotype analysis was performed. Results: Of the probands, 26.5% (9/34) from 7/28 unrelated families (25%) in the Jewish community of Rome harbored the known *BRCA2* c.7007G>C, p. (Arg2336Pro) variant (rs28897743). Genetic analysis of the four unrelated carriers revealed a shared haplotype, indicating a potential founder effect. The length of the haplotype might confirm the Roman community to be the oldest among Jewish communities in Europe. Conclusions: This study indicates the *BRCA*2 c.7007G>C variant found in the Jewish community of Rome to be a founder variant. Finally, we underline a pressing need to address the increased risk of carrying *BRCA* mutations among individuals with Jewish heritage, and to enhance genetic counseling and screening efforts in ethnic minorities that are not otherwise routinely reached.

## Simple Summary

Ethnical minorities can present higher rates of cancer susceptibility variants with a founder effect. This is the first study on *BRCA*1/2 analysis in breast and ovarian cancer cases in the Jewish community of Rome. A significant portion of probands with breast/ovarian cancer from the Jewish community of Rome harbored the heterozygous *BRCA2* c.7007G>C pathogenic variant. Haplotype analysis suggested a founder effect with remote origin.

## 1. Introduction

Universal genomic testing for cancer susceptibility genes appears to be a promising strategy for identifying high-risk individuals with a positive impact on cancer morbidity and mortality [1]. However, issues such as test reimbursement, human and economic resources, training of health professionals [1], and disparities in offering genetic testing remain [2]. Some ethnic minorities have been extensively studied for cancer susceptibility. For example, in Ashkenazi Jewish (AJ) population, three *BRCA1/2* (*BRCA*) pathogenic variants (PVs) with founder effect are responsible for 10% of breast cancers (BC) and 40% of ovarian cancers (OC) [3]. The US Comprehensive National Breast Cancer Network [4] identified AJ adults to be at higher risk for Hereditary Breast Ovarian Cancer (HBOC), and more recently, the National Health Service in England launched a national *BRCA* testing for adults of Jewish ancestry due to a higher probability of harboring a germline pathogenic variant (PV) [5]. Furthermore, the recent American Society of Clinical Oncology (ASCO) guidelines have developed recommendations for women with newly diagnosed BC from communities with increased prevalence of founder mutations [6]. The presence of a few specific variants has allowed for the rapid and low-cost screening of AJ populations. Screening of ethnic minorities has been conducted to identify high-risk groups in Europe [7], and a wide population study was also conducted in Israel among Sephardic and Asian Jewish groups without identifying any founder effect. However, a few prevalent variants have been included in the Israeli nationwide panel [8]. Currently, no information regarding *BRCA* status is available for the Jewish communities in Rome, which include the Roman Jews, one of the most ancient European communities settled after Jerusalem temple destruction, and the Sephardic Jews from Libya, who settled in Rome 50 years ago.

This study aimed to perform germline *BRCA* screening among women with BC and/or OC in this ethnic group, regardless of family history, while also examining possible founder variants and their functional effects.

## 2. Materials and Methods

### 2.1. Sample Selection and Statistical Analysis

Patients were recruited from Family Cancer Clinic, Department of Oncology at Umberto I University Hospital of Rome, the Outpatient of Jewish Hospital in Rome, and recruited through local Jewish journal advertisements between December 2016 and July 2024. Enrollment was proposed for individuals who self-identified as Jewish, had at least one Jewish parent, a history of BC and/or OC, and secondarily for first-degree family members after the identification of *BRCA* PVs. All participants were offered genetic counseling and screening for *BRCA* germline variants. Clinical data, including personal and family cancer history, age at onset, and pathological characteristics of BC/OC in at least three generations, were collected anonymously, and written consent forms and informative letters during genetic counseling were provided. Continuous variables were summarized using the median and interquartile range (IQR). Categorical variables were summarized using counts and percentages. The 95% confidence interval was calculated using Wilson’s exact method. To evaluate the association between categorical variables, we performed the chi-square test or Fisher’s exact test when appropriate. Statistical analyses were performed using R v4.0.4 (R Foundation for Statistical Computing, Vienna, Austria).

The protocol was approved by the Institutional Review Board ( approval number 636/16 protocol 5 November 2016).

### 2.2. BRCA Testing and Haplotype Analysis

#### 2.2.1. *BRCA* Testing

The QIAmp DNA Mini kit was used to extract Genomic DNA from whole blood samples on the Qiacube instrument (Qiagen, Milan, Italy). To quantify the extracted DNA, we used the Qubit dsDNA BR fluorometric assays (Life Technologies, Gaithersburg, MD, USA). The purity and quality of the extracted DNAs were assessed by using a spectrophotometer method and agarose gel. Only DNAs meeting specific requirements (OD260/280 ratio ≥ 1.7, concentration ≥ 15 ng/μL, and no degradation signals visible on agarose gel) were used for *BRCA* testing. *BRCA* status was assayed using the amplicon-based library preparation *BRCA* Devyser kit (Devyser, Stockholm, Sweden) that covers all the coding regions and the exons boundaries of *BRCA* genes, as previously reported [9]. Using the Qubit dsDNA HS fluorometric assays (Life Technologies, Gaithersburg, MD, USA), DNA libraries were quantified and processed via Next Generation Sequencing (NGS), using the Illumina MiSeq Reagent kit v2 (500-cycles) in paired-end reads mode (2 × 251 cycles) with FastQ only analysis workflow performed on the Illumina MiSeq^®^ NGS platform (Illumina, San Diego, CA, USA). Data analysis was performed to detect Single Nucleotide Variants, insertions/deletions (indels) and Copy Number Variations accounted in *BRCA* genes. Sequencing FastQ data were analyzed by the CE-IVD Amplicon Suite Software v3.7.0 (SmartSeq, Novara, Italy). The bioinformatic CNV prediction was used to analyze the coverage levels of the target regions across samples with the resolution of single exon. The variants were classified according to the American College of Medical Genetics and Genomics (ACMG) guidelines [10] and the *BRCA*-specific ClinGen ENIGMA expert panel recommendations [11]. The final variant classification was obtained by querying multiple online databases, including GnomAD (https://gnomad.broadinstitute.org/, last accessed 12 May 2025), 1000 Genomes (http://www.internationalgenome.org, last accessed 04 April 2025), ClinVar (http://www.ncbi.nlm.nih.gov/clinvar/, last accessed 10 May 2025), LOVD (https://www.lovd.nl/, last accessed 10 May 2025), ENIGMA (https://enigmaconsortium.org/, last accessed 12 May 2025), Franklin (https://franklin.genoox.com/clinical-db/home, last accessed 10 May 2025), and Varsome (https://varsome.com, last accessed 10 May 2025). The identified variants were reported in accordance with the Human Genome Variation Society nomenclature guidelines (https://varnomen.hgvs.org/ last accessed 10 May 2025.

#### 2.2.2. Haplotype Analysis

Genomic DNA was extracted using the MagPurix Blood DNA Extraction Kit and MagPurix Automatic Extraction System (Resnova, Rome, Italy). DNA quality and concentration was evaluated using a DeNovix Spectrophotometer (Resnova, Rome, Italy). Haplotype analysis was performed with SNP-array (single-nucleotide-polymorphism-array) technology (Illumina CytoSNP-850K v1.2) on genomic DNA from individuals 14A, 20A, 16A, and 2F (three probands and one unaffected member). Beeline Software v2.0.3 (Illumina) was used for data analysis. Phasing was achieved using theSHAPEIT v2.r900 software [12] with the 1000 Genomes Phase 3 dataset as a reference panel [13].

## 3. Results

### 3.1. Sample Selection

A total of 44 subjects (34 Roman Jews and 10 Sephardic Jews from Libya) from 38 unrelated families (28 and 10, respectively) were included. A total of 41 probands were screened for *BRCA* genes. Two patients, affected by BC and OC, respectively, had already tested and resulted positive, and one obligate carrier affected by BC was also included. The median age at screening was 64 years (IQR 55–70). Among the probands, thirty-nine had BC a history (median age at diagnosis 56, IQR 45.5–65), four had OC (median age at diagnosis 59, IQR 48.5–69), and one had both BC and OC (Table 1 and Table 2).

Most BC patients had invasive ductal carcinoma (33/40 probands, 82.5%), a luminal molecular phenotype (29/40 probands, 72.5%), and were at an early stage (stage I) at diagnosis (19/40 probands, 47.5%) (Table 3). All OC cases were of the high-grade serous carcinoma (HGSC) subtype, and 4/5 probands (80%) were diagnosed at stage III (Table 3).

### 3.2. BRCA Screening

Among the 38 unrelated families, molecular testing detected two BRCA*2* variants in 8/28 (28.6%) Roman Jewish families. The c.7007G>C, p. (Arg2336Pro) variant was found in heterozygosity in seven out of twenty-eight (25%) [95%CI (11.4–45.2)] unrelated families, for a total of 9/34 probands (26.5%). Seven probands were affected by BC (including two who had already tested positive) and two by OC (including one who had already tested positive) (Table 4). A second *BRCA*2 variant, c.7963C>T, p. (Gln 2655*) was found in 2 out of 34 BC probands (6%) from the same family (1/28, 3.5%) (Table 4). Only one side of this family belonged to the Jewish community of Rome, and no living family members were available for the segregation analysis. Based on family history, the identified variant probably originated from the non-Jewish side, specifically from Central Italy (Umbria and Tuscany). The c.7963C>T (p.Gln 2655*) has already been reported in individuals from Tuscany [14]. No *BRCA* PVs were found in patients of Libyan origin. Four variants of uncertain significance (VUS) were detected in the 34 Roman Jewish probands (Table 4).

Data on the members of the families carrying the 7007G>C *BRCA2* variant is provided in Table 5 and Table 6.

Segregation analysis of a family carrying the c.7963C>T variant revealed one obligate carrier for a total of four carriers, three affected and one unaffected.

The c.7007G>C, (rs28897743), p. (Arg2336Pro) variant in *BRCA2* can be classified as pathogenic according to the latest *BRCA2*-specific curation of the ACMG guidelines [10] by the ClinGen ENIGMA expert panel [11] with the PS3 supporting, PM3 strong, PM2 supporting, and PP3 supporting criteria.

The c.7963C>T variant is classified as pathogenic according to the American College of Medical Genetics and Genomics guidelines [10] and has been reported in accordance with the Enigma guidelines (Enigma.consortium.org).

Sanger sequencing confirmed the heterozygous variants in all carriers.

### 3.3. Haplotype Analysis

**Figure 1:**
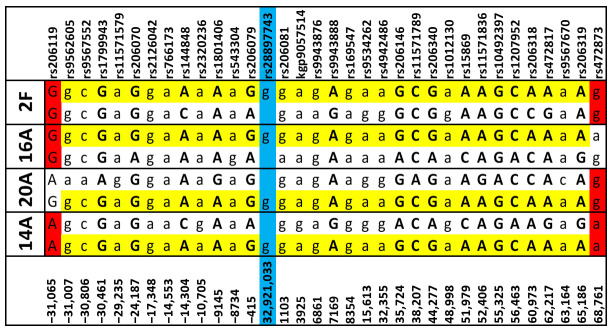
The segment includes the c.7007 G>C variant (highlighted in blue). Marker positions are indicated as base pair (bp) distances from the variant. Capital letters denote markers used by SHAPEIT for phasing; lowercase letters denote Illumina markers with imputed haplotype allocations. Red markers at the extremities indicate “hard breaks” (defined as the sites where any two subjects are homozygous for different alleles) between 14A and the others. The shared haplotype is highlighted in yellow. Markers with the same homozygous allele across all subjects are omitted.

The results are presented in Figure 1. The hard breaks at the extremities of the segment between subjects 14A and 2F demonstrated the interruption of a shared haplotype beyond these two points. However, the shared haplotype extended beyond these extremities for both 14A–20A and 14A–16A pairs (one-sided only). Accurately estimating their full length is challenging due to the decreasing accuracy of SHAPEIT for long haplotypes. Conversely, subjects 20A, 16A, and 2F potentially shared another haplotype extending far beyond both extremities. The shared haplotype length between 14A and 2F is only 96.2 kb, equivalent to roughly 0.1 cM in recombination frequencies.

## 4. Discussion

To our knowledge, this is the first report in Europe to describe a *BRCA2* founder variant in a non-Ashkenazi Jewish community. The high frequency of carriers of the c.7007G>C *BRCA2* variant among OC and BC probands (26.5%) might justify clinical *BRCA1/2* testing for all individuals of the community with personal or familial history of BC/OC. SNP genotyping of four selected subjects revealed a shared haplotype surrounding the c.7007G>C variant, suggesting that this segment represents a remnant of the ancestral chromosome of a common ancestor. More specifically, the shared haplotype length between individuals 14A and 2F was only 96.2 kb, which is equivalent to about 0.1cM in recombination distance. A rule of thumb in genetic genealogy is that if two individuals share a 1 cM segment of DNA, they likely share a most recent common ancestor roughly 100 generations ago, or about 2500 years in the past, for two people living today. Considering that the actual length of the shared segment between 14A and 2F is one-tenth of this value, it can be cautiously assumed that the common ancestor of this fragment could have lived more than 10,000 years ago.

In recent decades, research efforts to identify communities with increased cancer susceptibility and with relevant founder variants have been directed towards different geographical/ethnic groups. No *BRCA* screening among Italian Jewish communities has been reported, except for one study in which 107 healthy Roman Jewish subjects were genotyped for the three AJ *BRCA* founder variants, which reported no carriers [15]. The Jewish community of Rome has been a historically segregated group for nearly two millennia (albeit with a possible expansion after the expulsion of Sephardic Jews from Spain in 1492). Isolation within a walled ghetto erected in the Middle Ages continued until 1870, likely leading to endogamous marriages and potentially forming a unique genetic pattern [16].

The c.7007G>C *BRCA2* variant, identified with high prevalence in this community, has been reported only in a few carriers from worldwide *BRCA* screening studies [17], more specifically in the Balkans, the Mediterranean area, and in Israel [18,19,20,21,22]. This variant has been proposed in an Israeli nationwide *BRCA* screening program for founder/recurring variants [8,20].

Notably, our group reported that, in a single reference hospital in Rome, the c.7007G>C variant was found in 4/2351 patients [23]. Two are included in the present study, and two were non-Jewish individuals of Apulian origin. In the same geographic area, three further c.7007G>C carriers were identified: 2/2026 probands from population screening [24] and 1/95 harboring *BRCA* variants from 319 high-grade serous OC [25,26]. The Apulia region showed an increased percentage of the *BRCA1* c.5266dupC AJ founder variant, confirming the Jewish settlement in this area [24]. This could be a trace of the passage of Jewish people to Northern Europe through Italy (Apulia), as documented in [27]. Comparing the prevalence of the c.7007G>C variant in the Balkans and the Mediterranean area of Europe with our findings (25%), a potential migratory route of the Jewish community to Rome, possibly through Apulia, indicating ancient settlements and a later migration to Balkans, Greece and the Mediterranean area might be proposed.

Haplotype analysis in cases from these regions would be required to confirm a common origin. The available data suggest that the *BRCA2* c.7007G>C allele in the Jewish population of Rome might be older than the *BRCA1* c.5266dup AJ allele, which is one of the three founder mutations in AJ. The origin of the AJ *BRCA1* c.5266dup allele traces back to Scandinavia or northern Russia ~ 1800 years in the past. Its introduction into the AJ population is believed to have occurred approximately 400–500 years ago in Poland [28].

The *BRCA2* c.7007G>C is a missense variant in the last nucleotide of exon 13, demonstrated in the literature to promote abnormal splicing [29,30]. It is not yet definitively classified by Enigma. It lies in the *BRCA2* Ovarian Cancer Cluster Region (OCCR) [31,32] and has been associated with Fanconi anemia in newborns bearing biallelic *BRCA2* PVs [33,34]. It can be classified as pathogenic according to the ClinGen/ENIGMA *BRCA* expert panel recommendations for the ACMG guidelines (PS3 strong, PM3 strong, PM2 supporting, and PP3 supporting criteria) [10,11].

Cancer prevention and management strategies are extremely complex, and clinical, economical, and social factors need to be taken into consideration, while also balancing nation-wide standardization with individual patient needs [35]. Collaborative efforts to propose standardized approaches on the integration of *BRCA* testing on BC screening/management have been undertaken in Italy [36]. Our study might suggest how, in some areas, specific ethnic minorities might benefit from dedicated programs within broader national and international protocols.

## 5. Conclusions

The *BRCA2* c.7007G>C variant found with high prevalence in the Roman Jewish BC/OC families appears as a possible founder variant. The analysis should be extended to more individuals, both Roman Jews and from different ancestries, to provide a more precise estimation of the most recent common ancestor and unveil the history of the ancestral allele. More extended segregation analysis on the index families might strengthen the validity of the results and provide longer genealogic tracing. The number of Sephardic families in the study limits possible inferences and comparisons concerning this group.

Our findings support the role of population-specific clinical and molecular screening approaches for ethnic minorities that are not otherwise reached. This can result in more appropriate cancer risk management, and provide unique insights into population genetics and human migration history.

## Figures and Tables

**Table 1:** Characteristics of subjects enrolled.

	Total	Roman Jews	Sephardic Jews
Female n (%) **Gender** Male n (%)	44 (100)	34 (77)	10 (23)
	0 (0)	0 (0)	0 (0)
**Age at screening** Median (IQR)	64 (55–70)	65.5 (55–75.25)	61.5 (53.25–69.25)
**Breast cancer cases** n (%)	39 (89)	31 * (79)	8 (21)
**Ovarian cancer cases** n (%)	4 (9)	3 (75)	1 (25)
**Breast and ovarian cancer cases** n (%)	1 (2)	0 (0)	1 (100)

* n = 6 metachronous bilateral breast cancer; n = 1 synchronous bilateral breast cancer.

**Table 2:** Median age at diagnosis.

	Subjects Enrolled (n = 44)	Roman Jews	Sephardic Jews
**Median Age at diagnosis** (IQR)	56 (47.25–65)	57.5 (47.25–65.5)	51.5 (46.5–56)
**Median Age of BC**(IQR)	56 (45.5–65)	58 (45–65)	48 (46–55)
**Median Age of 2° BC**(IQR) *	76 (62–80)	76 (62–80)	
**Median Age of OC**(IQR)	59 (48.5–69)	49 (NA-NA)	60.5 (NA-NA)

* Six cases of second breast cancer (n = 1 synchronous bilateral breast cancer; n = 4 metachronous bilateral breast cancer; n = 1 metachronous ipsilateral breast cancer). BC = breast cancer. OC = ovarian cancer. NA = not applicable.

**Table 3:** Tumor characteristics and genotypes of 44 subjects enrolled.

Breast Cancer (n = 40) ^			2° Breast Cancer (n = 7) #	Ovarian Cancer (n = 5) ^		
**Age at Diagnosis**	**56 (45.5–65)**		**76 (62–80)**	**Age at Diagnosis**	59 (48.5–69)	
**Histotype ** **N (%)**	IDC	33 (82.5)	6 (86)	**Histotype ** **N (%)**	Serous	5 (100)
	ILC	2 (5)	0 (0)			
					Mucinous	0 (0)
	DCIS	4 (10)	1 (14)			
					Others	0 (0)
	LCIS	1 (2.5)	0 (0)			
**Subtype ** **N (%)**	LUMINAL	29 (72.5)	5 (72)	**Grade (Serous OC) ** **N (%)**	Low grade	0 (0)
	HER2	1 (2.5)	0 (0)			
	TN	4 (10)	1 (14)		High grade	5 (100)
	NA	6 (15)	1 (14)			
**Stage ** **N (%)**	0 (pTis)	5 (12.5)	1 (14)	**Stage ** **N (%)**	I	0 (0)
					II	1 (20)
	I	19 (47.5)	1 (14)			
	II	7 (17.5)	3 (43)			
					III	4 (80)
	III	2 (5)	0 (0)		IV	0 (0)
	IV	2 (5)	1 (14)			
					NA	0 (0)
	NA	5 (12.5)	1 (14)			
**Genotype ** **N (%)**	Carriers	9 * (22.5)	3 (43)	**Genotype ** **N (%)**	Carriers	2 * (40)
	WT	28 (70)	4 (57)			
					WT	2 (40)
	VUS°	3 (7.5)	0 (0)		VUS°	1 (20)

^ One subject showed breast and ovarian cancers in both tumors. * Two pathogenic variants were identified. -c.7007G>C in 9 patients, 7 affected by BC and 2 by OC. -c.7962 C>T in 2 patients affected by BC. # n = 6 metachronous bilateral breast cancers; n = 1 synchronous bilateral breast cancer. Abbreviations: DCIS = ductal carcinoma in situ; IDC = invasive ductal carcinoma; ILC = invasive lobular carcinoma; LCIS = lobular carcinoma in situ; NA = not applicable; OC = ovarian cancer; TN = triple negative; VUS = uncertain significance variants; WT = wild type.

**Table 4:** Prevalence of *BRCA* PV (pathogenetic variants) and VUS (variant of uncertain significance) among enrolled subjects.

	Variants Identified	N° of Families Carriers (%/28)	N° of Subjects Carriers (%/34)	Type of Tumor	
				BC	OC
**PV ** ***BRCA2***	**c.7007G>C**	**7 (25) °**	9 * (26.5)	7	2
	c.7962C>T	1 (3.5) °	2 ^ (6)	2	0
**VUS ** ***BRCA 1***	c.3691 T>C	1 (3.5)	1 (3)	1	0
	c.3367 G>T	1 (3.5)	1 (3)	0	1
**VUS ** ***BRCA 2***	c.1259A>G	1 (3.5)	1 (3)	1	0
	c.280C>T	1 (3.5)	1 (3)	1	0

PV = pathogenic variant; VUS = uncertain significance variant; BC = breast cancer; OC = ovarian cancer. **° ***BRCA2* PVs were detected in 8/28 (28.6%) families of Jewish Rome origin.* Nine subjects belonging to seven unrelated families tested positive for c.7007G>C variant. ^ Two subjects belonging to the same family tested positive for c.7962C>T variant.

**Table 5:** Characteristics of the seven unrelated families carrying the c.7007G>CA *BRCA2* PV.

	Total N°	Median Per Family (IQR)		A/U	Total N °	Median Per Family (IQR)
**Subjects**	179	25 (15–33)		**A**	44	5 (3–9)
				**U**	135	20 (13–25)
***BRCA *** **m subjects ^**	38	4 (4–8)		**A**	21	2 (2–5)
				**U**	17	2 (1–3)
**WT subjects #**	29	4 (3–5)		**A**	4	1 (0–1)
				**U**	25	4 (2–4)
**Tested subjects ** **(carriers)**	12	2 (1–2)		**A**	12	2 (1–2)
				**U**	0	0 (0–0)
**Tested subjects ** **(non carriers)**	5	1 (0–1)		**A**	4	1 (0–1)
				**U**	1	0 (0–0)
**Obligate carriers**	8	1 (0–2)		**A**	7	1 (0–2)
				**U**	1	0 (0–0)
**Segregation analysis (carriers)**	18	2 (1–3)		**A**	2	0 (0–1)
				**U**	16	2 (1–3)
**Segregation analysis (non carriers)**	24	4 (2–4)		**A**	0	0 (0–0)
				**U**	24	4 (2–4)
**Unknown Genotype**	112	15 (12–23)		**A**	19	1 (1–5)
				**U**	93	14 (10–19)

° Total number= cumulative sum of participants among seven families. ^ BRCAm subjects = sum of tested subjects (carriers), obligate carriers, segregation analysis (carriers). # WT subjects= sum of tested subjects (non-carriers), segregation analysis (non-carriers). A = affected; U = unaffected; *BRCA*m = carriers of *BRCA* pathogenic variants; WT = wild type.

**Table 6:** Tumor characteristics and genotype of affected subjects in the seven unrelated families tested positive for the *BRCA2* c.7007G>C PV.

	Total N° of Subjects ° [Median Per Family (IQR)]	Age at Diagnosis Median (IQR)	Total N° of Carriers ° [Median Per Family (IQR)]	Total N° of Non-Carriers ° [Median Per Family (IQR)]	Total N° Untested Subjects ° [Median Per Family (IQR)]
Affected subjects *	44 [5 (3–9)]	62 (51–69.5)	21 [2 (2–5)]	4 [1 (0–1)]	19 [2 (1–5)]
**Breast cancer ^**	29 [5 (0–7)]	60 (48.75–70)	13 [1 (0–4)]	4 [1 (0–1)]	12 [1.5 (0–3)]
**Male breast cancer**	2 [0 (0–1)]	65 (NA)	2 [0 (0–1)]	0	0
**Ovarian cancer**	7 [1 (0–2)]	55 (49–62)	5 [0 (0–1)]	0	2 [0 (0–1)]
**Prostate cancer**	2 [0 (0–1)]	69.5 (NA)	1 [0 (0–0)]	0	1 [0 (0–0)]
**Pancreatic cancer**	1 [0 (0–0)]	76 (NA)	1 [0 (0–0)]	0	0
**Melanoma**	2 [0 (0–1)]	62 (NA)	0	0	2 [0 (0–1)]
**Gastric cancer**	3 [0 (0–1)]	61 (NA)	2 [0 (0–1)]	0	1 [0 (0–0)]
**Colorectal cancer**	2 [0 (0–1)]	49.5 (NA)	1 [0 (0–0)]	0	1 [0 (0–0)]
**Malignant glioma**	1 [0 (0–0)]	70 (NA)	0	0	1 [0 (0–0)]
**Thyroid cancer**	1 [0 (0–0)]	50 (NA)	1 [0 (0–0)]	0	0

° Total number = cumulative sum of participants among seven families. * Five cases of multiple tumors. (1) BC (56)/GC (67). (2) MBC (66)/PR (74)/PA (76). (3) BC (50)/OC (55). (4) OC (42)/GC (61). (5) TC (50)/BC (55). ^ Three cases of bilateral BC and one case of ipsilateral BC. BC = breast cancer; GC = gastric cancer; MBC = male breast cancer; PA = pancreatic cancer; PR = prostate cancer; TC = thyroid cancer.

## Data Availability

The complete dataset is not publicly available because of privacy concerns and the ethical restrictions imposed by the Ethical Committee. Reasonable requests for access to the full dataset can be directed to the corresponding author. Access will be granted upon approval from the Data Protection Officer of the “Fondazione Policlinico Universitario A. Gemelli” IRCCS in Rome, IRCCS Fondazione Santa Lucia, and Fondazione Pisana per la Scienza, Pisa, Italy.

## Abbreviations

The following abbreviations are used in this manuscript:

ACMG	American College of Medical Genetics and Genomics
AJ	Ashkenazi Jews
BC	Breast cancer
HBOC	Hereditary breast/ovarian cancer
HGSC	High-grade serous cancer
IQR	Interquartile range
OC	Ovarian cancer
PV	Pathogenic variant
VUS	Variant of uncertain significance
